# Prenatal Chromosomal Microarray Analysis: Does Increased Resolution Equal Increased Yield?

**DOI:** 10.3390/genes14081519

**Published:** 2023-07-25

**Authors:** Anastasios Mitrakos, Konstantina Kosma, Periklis Makrythanasis, Maria Tzetis

**Affiliations:** Laboratory of Medical Genetics, Medical School, National and Kapodistrian University of Athens, St. Sophia’s Children’s Hospital, 11527 Athens, Greece; kokosma@med.uoa.gr (K.K.); pmakryth@med.uoa.gr (P.M.)

**Keywords:** prenatal diagnosis, amniotic fluid, chorionic villi, array comparative genomic hybridization, aCGH

## Abstract

Chromosomal microarray analysis (CMA) is considered a first-tier test for patients with developmental disabilities and congenital anomalies and is also routinely applied in prenatal diagnosis. The current consensus size cut-off for reporting copy number variants (CNVs) in the prenatal setting ranges from 200 Kb to 400 Kb, with the intention of minimizing the impact of variants of uncertain significance (VUS). Very limited data are currently available on the application of higher resolution platforms prenatally. The aim of this study is to investigate the feasibility and impact of applying high-resolution CMA in the prenatal setting. To that end, we report on the outcomes of applying CMA with a size cut-off of 20 Kb in 250 prenatal samples and discuss the findings and diagnostic yield and also provide follow-up for cases with variants of uncertain significance. Overall, 19.6% (49) showed one or more chromosomal abnormalities, with the findings classified as Pathogenic (P) or Likely Pathogenic (LP) in 15.6% and as VUS in 4%. When excluding the cases with known familial aberrations, the diagnostic yield was 12%. The smallest aberration detected was a 32 Kb duplication of the 16p11.2 region. In conclusion, this study demonstrates that prenatal diagnosis with a high-resolution aCGH platform can reliably detect smaller CNVs that are often associated with neurodevelopmental phenotypes while providing an increased diagnostic yield, regardless of the indication for testing, with only a marginal increase in the VUS incidence. Thus, it can be an important tool in the prenatal setting.

## 1. Introduction

Chromosomal microarray analysis (CMA) has been considered a first-tier test for the detection of copy number variants (CNVs) in patients with developmental disabilities and congenital anomalies for more than a decade [1] due to the increased diagnostic yield over conventional cytogenetics as well as other advantages, including faster turnaround times and increased sensitivity. However, the adoption of the technique in the prenatal setting has been slower [2], mainly due to variants of uncertain clinical significance (VUS) that can increase parental anxiety and possibly lead to unwarranted terminations of pregnancies [3].

To aid the prenatal application of CMA, professional societies worldwide have released guidelines providing consensus instructions for evaluating and reporting prenatal CMA results [4,5,6,7,8,9]. The current consensus size cut-off ranges from 200 Kb to 400 Kb, while it has even been suggested that a cut-off of 500 Kb for deletions and 1 Mb for duplications should be used in the prenatal setting [8]. Other recommendations include that the reporting of findings should be limited to those that have a clearly documented genotype-to-phenotype correlation [10]. In addition to VUS, the incomplete penetrance and variable expressivity of several variants might also explain why higher-resolution platforms have, at large, been avoided in the prenatal setting.

On the other hand, higher-resolution platforms could potentially offer advantages by increasing diagnostic yield in the prenatal setting; however, their impact has not been evaluated due to the limitations explained above. The aim of this study is to investigate the feasibility and impact of applying high-resolution CMA prenatally.

To this end, we report on the outcomes of applying a high-resolution CMA platform in the prenatal setting and discuss the findings, diagnostic yield and follow-up for cases with variants of uncertain significance.

## 2. Materials and Methods

In total, 219 couples were referred to the Laboratory of Medical Genetics of the National and Kapodistrian University of Athens for prenatal CMA. The average maternal age of the couples was 34.9 years (range 19–46 years). For all the cases, either chorionic villus sampling or amniocentesis was performed. The indications for CMA testing included fetal structural abnormalities identified through ultrasonographic screening, increased nuchal translucency (≥3.5 mm), previous pregnancy or offspring with a chromosomal abnormality or CNV or a known parental chromosomal abnormality or CNV, as well as advanced maternal age (AMA) and parental anxiety. Additionally, CMA testing was offered to couples requesting the test after having been subjected to invasive sampling to test for monogenic disorders (recessive or dominant).

A total of 250 samples were studied (143 AF, 107 CVS), of which 141 (56.4%) were male and 109 (43.6%) were female. Maternal cell contamination was excluded for all the samples via the use of polymorphic STR markers.

From the 250 fetuses that were subjected to CMA, 15.2% (38) had AMA as their indication for testing, 23.6% (59) had a family history of a previous child or pregnancy with a chromosomal abnormality or parent with a balanced chromosomal translocation, 48% (120) were due to parental anxiety or as an adjunct to invasive prenatal testing for a monogenic disorder, 12.8% (33) had abnormal ultrasound findings ranging from mild to severe and/or positive biochemical screening (PAPP-A) values as their only indication for testing.

Genomic DNA was extracted from uncultured amniotic fluid (*n* = 143) or chorionic villi samples (*n* = 107), as well as from parental peripheral blood samples, using the commercially available QiAmp DNA Mini kit (Qiagen, Hilden, Germany), according to the manufacturer’s instructions. The quality and quantity of the DNA samples were determined using the NanoDrop ND-1000 UV-Vis spectrophotometer.

High-resolution aCGH analysis was performed with the Agilent Human Genome 4 × 180 k G3 CGH + SNP microarray platform, which contains ~120,000 CGH probes with a median probe spacing of ~25 Kb, as well as ~60.000 SNP probes that provide detection of copy neutral loss of heterozygosity (CN-LOH) with a resolution of ~5–10 Mb (Agilent Technologies, Santa Clara, CA, USA). In specific cases with known aberrations below the threshold of the 4 × 180 K platform, a higher-resolution platform (2 × 400 K G3 CGH + SNP, Agilent Technologies, Inc., Santa Clara, CA, USA) was used containing ~292,000 CGH with a 7.2 Kb overall median probe spacing and ~119,000 SNP probes.

The protocol used for aCGH and analysis of the resulting data with CytoGenomics version 5.0.2 software (Agilent Technologies, Santa Clara, CA, USA) was as previously described [11,12]. The ADM-1 aberration detection method was utilized with a log2ratio threshold of 0.25 for both the deletions and duplications, with the minimum number of probes required for a call set to 4. The aberrations flagged by the software were also manually inspected to eliminate any false positive calls. For the location of genes in the deleted or duplicated genomic segments and their prevalence in the population, the UCSC genome browser (http://genome.ucsc.edu/ (accessed on 28 March 2023)) and the Database of Genomic Variants (http://projects.tcag.ca/variation/ (accessed on 28 March 2023); human genome build 19) were used. The variant classification was based on an evaluation of the genomic content, overlap with an established or predicted haploinsufficient or triplosensitive region, the number of genes included in the aberrant segment, the inheritance pattern and data from the literature, public and in-house databases, providing genotype to phenotype correlation from postnatal samples with similar aberrations. Statistical analysis of the results was performed in order to determine the diagnostic yield and rates of pathogenic and likely pathogenic variants and variants of uncertain significance.

Signed informed consent was obtained from all the couples.

## 3. Results

Overall, 201 fetuses (80.4%) had a normal karyotype, and 49 (19.6%) showed one or more chromosomal abnormalities, with 20 deletions and 43 duplications being identified overall.

The smallest aberration detected was a 32 Kb duplication of the 16p11.2 region, and the largest was a 22.8 Mb duplication of the 7q33-q36.3 region. With the use of the 2 × 400 K G3 CGH + SNP platform, an even smaller pathogenic deletion of only 3.8 Kb, including the *WAS* gene, was detected.

The findings were classified as pathogenic (P) or likely pathogenic (LP) in 39 cases (15.6%) (Table 1) and as variants of uncertain clinical significance (VUS) in 10 cases (4%) (Table 2). When excluding the cases with known familial aberrations, pathogenic and likely pathogenic findings were observed in 12% of the cases.

In six cases, CMA revealed two pathogenic chromosomal abnormalities in the same embryo (Table 1).

An analysis of the parental samples revealed that the findings had occurred de novo in five cases, while they had been maternally inherited in six cases (two from the same family) and paternally inherited in five cases (two from the same family).

For the cases that revealed a P, LP or VUS finding, 34.6% had family history as their indication for testing (including previous child or previous pregnancy or parent with chromosomal abnormality and/or balanced translocation), 53% had advanced maternal Age or performed testing due to parental anxiety and 12.2% had abnormal ultrasound findings and/or abnormal biochemical screening results.

Of the 33 cases with an abnormal ultrasound and/or biochemical screening, 18.5% showed a clinically significant CMA result.

Out of 125 cases that requested CMA due to AMA or due to parental anxiety, 20% had a P or LP finding.

## 4. Discussion

Here, we report on the outcomes of applying prenatal CMA analysis with a high-resolution platform. Overall, P or LP aberrations were found in 15.6% of the patients. When excluding the cases with known familial aberrations, the diagnostic yield was 12%. The VUS incidence overall was 4%.

Breman and colleagues reported a diagnostic yield of 4.2% with the use of CMA in over 1000 fetuses [13], after excluding cases with known or familial chromosomal abnormalities. The VUS incidence in the same study was 1.6%. The study spanned several years and, thus, CMA was performed on various custom-designed platforms. The lowest-resolution platform was based on BAC probe arrays and targeted 40 genomic disorders, while the newest platform had a backbone resolution of ~100 Kb via the use of oligonucleotide arrays.

When grouping patients by indication, an increase in the diagnostic yield is expected in cases with abnormal prenatal ultrasonographic and/or biochemical screening. In a recent study by Tanner et al. [14] targeting only high-risk pregnancies with a 4 × 180 K CGH microarray platform with comparable resolution to ours, the diagnostic yield reported was 15.1% overall and increased to 20% in the case of fetuses with multiple structural abnormalities. The diagnostic yield was lowest in the group with intrauterine growth restriction (IUGR) as the sole indication for testing (5.7%). In our study, a pathogenic or likely pathogenic finding was detected in 18.5% of the high-risk cases with abnormal ultrasound and/or biochemical screening.

A more recent report by Lovrecic et al. [15] using the Agilent SurePrint G3 Unrestricted CGH ISCA v2 8 × 60K microarray, which provides a practical resolution of ~100 Kb, revealed a diagnostic yield of 10.0% in a cohort with ultrasonographic anomalies. Of those, the cases with multiple congenital anomalies showed the highest diagnostic rate of 16.7%, with only 6.3% of cases with isolated IUGR revealing a pathogenic CNV.

Due to the relatively small number of cases with abnormal ultrasound findings in our cohort, an estimate of the diagnostic yield for this group would be inaccurate; however, literature reports confirm that the highest diagnostic yield is observed in cases with multiple congenital anomalies.

The diagnostic yield in fetuses with increased nuchal translucency as their only indication for testing does not appear to be strongly correlated with P or LP findings in CMA, as discussed in a retrospective multicenter study by Egloff and colleagues [16]. In this study, the authors found pathogenic CNVs in only 2.7% of fetuses with nuchal translucency ≥3.5 mm for which rapid aneuploidy screening was negative, while only 1.3% of the cases had variants classified as VUS. All the studies included used oligonucleotide microarrays with variable resolution.

Higher-resolution CMA can identify smaller aberrations (>50 Kb in this study) that would be missed by lower-resolution platforms and has the potential to provide a diagnosis and aid couples in making informed reproductive decisions, regardless of the presence of ultrasonographic and/or biochemical screening markers. However, increased resolution can result in higher rates of VUS detection, which can add a psychological burden to the family and the attending physician, as well as potentially lead to unwarranted terminations of pregnancy.

When comparing the VUS incidence between a BAC and an oligonucleotide platform, Hillman et al. found an 8% increase in VUS [17]. However, these estimates vary greatly in the literature, with a recent cohort study showing that the prevalence of VUS after CMA with a high-resolution aCGH platform was 5.8%, and most of them were associated with a known phenotype that could be ascertained prenatally [18].

In a meta-analysis comparing the use of prenatal CMA and conventional karyotyping [19], Hillman and colleagues reported an increase of 4.1% in the diagnostic rate compared to conventional karyotyping when the indication for testing was abnormal fetal ultrasonographic findings. The rate of detection of VUS for the same group of patients was 2.1%. A lower rate of VUS (1.4%) was reported when the analysis included all the indications for testing.

The incidence of VUS in our study was, as expected, higher compared to the data from lower-resolution platforms, accounting for 4% of the cases.

A large study by Shaffer and colleagues that included over 5000 cases [20] determined an overall detection rate of clinically significant copy number alterations of 5.3%, which increased to 6.5% in cases with abnormal ultrasonographic findings. The incidence of VUS was 4.2%; however, after parental testing, if only de novo variants were considered, the rate was reduced to 0.39%. This approach was also utilized in some of the cases in our study, as the inheritance pattern of the findings can significantly impact their classification and management. As a rule of thumb, variants inherited from healthy parents are of lower risk; however, the final interpretation/counseling should also take into consideration that certain CNVs show incomplete penetrance and/or variable expressivity, further complicating the interpretation of results.

An important tool to minimize the impact of VUS, especially when using a high-resolution platform for prenatal testing, is a well-maintained in-house database with both prenatal and postnatal samples tested on the same microarray platform, as this can be an invaluable resource for small, rare aberrations for which there is not enough information in public databases, thus assisting with genotype to phenotype correlation and subsequent counselling without relying exclusively on published data or ultrasonographic and biochemical findings.

Gross ultrasound findings are more commonly associated with syndromic conditions arising from large chromosomal aberrations. However, smaller aberrations, which are often associated with neurodevelopmental syndromes, rarely give few, if any, ultrasonographic indications.

Out of the 39 P/LP findings, 6 were below 200 Kb (15%), which is the recommended minimum resolution for prenatal microarray testing and the most commonly applied threshold and, thus, would not have been detected by a lower-resolution platform. Additionally, of the 13 VUS variants, 6 were above the 200 Kb threshold (46%). These data suggest that higher-resolution analysis should not significantly increase VUS rates while at the same time offering a modest increase in the diagnostic yield.

Another benefit of the CGH + SNP platform, besides higher resolution, is the ability to detect regions of homozygosity (ROH), which may derive from consanguinity or uniparental disomy (UPD), as well as polyploidies (e.g., triploidy, tetraploidy) and haploidy via the incorporation of SNP probes in each array in addition to the CGH probes. Furthermore, the addition of SNP probes to the microarray can increase the mosaicism detection threshold. No region of UPD with clinical significance was detected in our cohort.

Below, we discuss the most interesting pathogenic, likely pathogenic and unknown significance variants found in our cohort and provide follow-up for cases with VUS where available.

### 4.1. Cases with Pathogenic Findings

Three cases with 16p13.11 duplications were identified (Table 1, cases 22, 23, 24), with two of them belonging to the same family and being paternally inherited. The 16p13.11 microduplication has been associated with several neurodevelopmental phenotypes, including ADHD, ASD, DD and ID, with variable phenotypic severity and penetrance [21]. The pathogenetic mechanisms and the genes responsible remain largely unknown, with a recent study establishing a 643 Kb region as the smallest region of overlap (SRO) for the microduplication syndrome [22]. Several cases are reported in the ClinVar and DECIPHER databases; thus, the variant was classified as pathogenic.

A male fetus was diagnosed with harboring a deletion of Xp11.23, which included MAOA, *MAOB* and *NDP* genes. Single nucleotide variants of the *NDP* gene cause Norrie disease, a rare X-linked disorder characterized by bilateral congenital blindness. Intragenic deletions, including NDP and the adjacent *MAOA*, *MAOB* and *EFHC2* genes, have been identified in about 15% of ND patients and have been associated with additional clinical features, such as microcephaly, growth delay, mental retardation and stereotypical movements [23], with significant phenotypic variability.

A deletion of Xq28 was also identified in a male fetus, including the *OPN1LW*, *OPN1MW2*, *OPN1MW*, *OPN1MW3*, *TEX28* and *TKTL1* genes. Pathogenic variants in the *OPN1LW*/*OPN1MW* gene cluster, including deletions, are responsible for a range of mild to severe visual impairments with color deficiencies [24].

The variants in genes related to Rett/Angelman syndrome phenotypes, including *MECP2*, *CDKL5*, *FOXG1*, *UBE3A*, *SLC9A6* and *TCF4*, present unique challenges in interpretation, so much so that very recently, gene-specific guidelines have been released to aid with interpretation [25]. In a male fetus, a duplication of 14q12 was identified that included part of the *FOXG1* gene. Pathogenic variants in *FOXG1* lead to the congenital variant of Rett syndrome (#613454), a severe neurodevelopmental disorder with features of classic Rett syndrome but earlier onset in the first months of life. The variant was classified as pathogenic, as the duplication breakpoints mapped inside the gene.

A small 83 Kb duplication in chromosome 6 including the *FOXCUT* and *FOXC1* genes was detected. *FOXC1* heterozygous variants cause Axenfeld–Rieger syndrome type 3 (#602482), an autosomal dominant multiple congenital anomalies disorder. Similarly, the variant was classified as pathogenic, as the duplication breakpoints were inside the gene.

A 99 Kb duplication of Xq25 spanning exons 13–16 of the *GRIA3* gene was identified during prenatal testing of a female and, subsequently, a male fetus from the same family. The *GRIA3* gene is an established haploinsufficient gene associated with X-linked syndromic intellectual developmental disorder (#300699). Parental testing showed that the duplication was maternally inherited and was also present in the couple’s daughter. Missense variants, deletions and translocations causing gene disruption have been reported to result in X-linked mental retardation phenotypes in male patients [26], while female carriers are usually asymptomatic.

A very small deletion of just 3.8 Kb in the Xp11.23 region, which included exons 2–7 of the *WAS* gene, was identified in a male fetus with known family history with the use of a very high-resolution aCGH platform (2 × 400 K G3 CGH + SNP, Agilent Technologies). Variants of the *WAS* gene lead to Wiskott–Aldrich syndrome, an X-linked recessive immunodeficiency characterized by thrombocytopenia, eczema and recurrent infections (#301000).

### 4.2. Cases with Likely Pathogenic Findings

A 180 Kb duplication of 2q36.3 with breakpoints inside the *TRIP12* gene was identified in one case (Table 1, Case 30). *TRIP12* is a member of the E3 ubiquitin ligases with involvement in important cellular processes and signaling pathways, including cell homeostasis, gene expression and cancer, with implications in cell cycle progression and maintenance of genome integrity [27]. Additionally, *TRIP12* is an established haploinsufficient gene with variants associated with Clark–Baraitser syndrome, an autosomal dominant intellectual developmental disorder (#617752). Specifically, truncating and missense variants, as well as one translocation, have been reported in patients with Clark–Baraitser syndrome [28]. The variant was classified as likely pathogenic.

In one case with a positive biochemical screening PAPP-A result, we identified a 286 Kb duplication of 3p26.3-p26.2, which included the *CNTN4* gene (Table 1, Case 31). The gene encodes for the Contactin-4 protein, a glycosylphosphatidylinositol-anchored neuronal membrane protein that is important for the early growth of developing axons and the maintenance of adult neuronal networks [29]. Variants that disrupt *CNTN4* have been associated with autism spectrum disorder (ASD) [30,31] as well as milder phenotypes, including speech delay, cognitive impairment and neurobehavioral phenotypes [29].

In four cases (Table 1, Cases 12, 19, 20, 21), a duplication of the 15q13.3 chromosomal region was identified. All the duplications involved the *CHRNA7* gene, and cases 19, 20 and 21 additionally included the *OTUD7A* gene. Although small duplications that include the *CHRNA7* gene are frequently detected in microarray studies and have been associated with a wide spectrum of neurodevelopmental phenotypes, including ASD, ID/DD, ADHD, OCD and epilepsy, their relatively high prevalence in the general population makes their interpretation difficult, even more so in the prenatal setting [32]. CNV data from 12,252 mother–father–child trios from the Norwegian Mother, Father, and Child Cohort Study (MoBa) revealed a higher-than-expected prevalence for 15q13.3 microdeletions/microduplications of about 1 in 2500, associating these variants with lower penetrance and/or milder clinical presentation than previous estimates and even suggesting the possible benign nature of smaller duplications [33]. Case 19, which had the larger 1.57 Mb duplication, proceeded with the termination of the pregnancy, while cases 12, 20 and 21 continued with the pregnancy and reported normal developmental milestones at ages 5 years, 18 months and 8 years, respectively.

A duplication of Xq28 including the *AFF2* gene was detected in a male fetus referred for prenatal chromosomal microarray analysis due to ultrasonographic findings of a ventricular septal defect and persistent left superior vena cava. The gene is associated with intellectual developmental disorder 19 (#309548) and, thus, the finding is not related to the reason for referral. The variant was classified as likely pathogenic as it includes part of an established haploinsufficient gene.

### 4.3. Cases with Variants of Uncertain Significance (VUS)

In two cases (Table 2, Cases 1–2), a deletion including the *LITAF* gene was identified. This gene encodes for the lipopolysaccharide-induced tumor necrosis factor, a transcription factor implicated in cytokine signaling and enriched in the peripheral nerves and Schwann cells [34]. Single nucleotide variants in this gene have been identified in patients with Charcot–Marie–Tooth type 1C disease (CMT1C), a demyelinating peripheral neuropathy inherited in an autosomal dominant manner [35]. The variant was classified as VUS as there are no reports of a deletion leading to the phenotype in the literature; however, there is one case in the ClinVar database with more than 80% overlap reported as pathogenic. These cases requested CMA due to parental anxiety and did not present abnormal findings in ultrasonographic and/or biochemical screening. Case 2 was followed up and resulted in the birth of a healthy neonate.

In one case, a 230 Kb duplication of 6q26 was identified that included the *PARK2* gene. CNVs in this gene have been reported as a risk factor for ADHD [36]. The finding was maternally inherited and, as such, was classified as a VUS. The pregnancy resulted in the birth of a healthy neonate with no clinical findings at the age of 6 years.

CNVs associated with neurodevelopmental disorders cannot be detected prenatally with ultrasonographic or biochemical screening. These CNVs may be smaller in size and situated in not well-defined areas of CNV-causing syndromes for which lower resolution aCGH prenatal testing platforms contain enough probes. Therefore, high-resolution aCGH analysis, as is becoming evident from the different cases reported herein, can help elucidate those neurodevelopmental disorder cases.

The main limitation of this study lies in its non-randomized retrospective nature, resulting in the inclusion of several familial cases in our cohort. To correct this, the diagnostic yield rate has also been calculated after the exclusion of familial cases.

## 5. Conclusions

In conclusion, we have provided evidence that prenatal diagnosis with a high-resolution aCGH platform can provide increased diagnostic yield, regardless of the indication for testing. Knowledge of the pathogenicity of CNVs has grown exponentially in recent years and is expected to increase further. This results in the VUS incidence being only slightly higher than in lower-resolution platforms. This effect can be further mitigated by parental sample testing and in-house databases. It is, thus, expected that the implementation of higher-resolution CMA for prenatal diagnosis will increase in the near future.

## Figures and Tables

**Table 1:** Cases with pathogenic/likely pathogenic variants (TOP: termination of pregnancy).

	Sample Type	Sex	Indication for Testing	Molecular Karyotype (ISCN)	Size	Classification	Associated Conditions	Follow-Up
1	CVS	F	PARENTAL ANXIETY	arr[GRCh37] 14q13.1q13.2 (34,857,622_36,420,214)×1 dn	1.56 Mb	PATHOGENIC		N/A
2	AF	M	PARENTAL ANXIETY	arr[GRCh37] 15q11.1q11.2 (20,416,244_23,217,514)×1	1.95 Mb	PATHOGENIC	15q11.2 Deletion syndrome (#615656)	N/A
3	AF	F	FAMILY HISTORY	arr[GRCh37] 15q11.1q11.2 (20,575,646_23,300,287)×1	2.7 Μb	PATHOGENIC	15q11.2 Deletion syndrome (#615656)	N/A
4	CVS	M	FAMILY HISTORY	arr[GRCh37] 15q11.1q13.3(20,190,548_33,528,589)×1 mat arr[GRCh37] 22q11.1q11.21(16,486,086_20,311,763)×3 mat	13.34 Mb/3.8 Mb	PATHOGENIC PATHOGENIC	15q11.2 Deletion syndrome (#615656)	N/A
5	CVS	F	PARENTAL ANXIETY	arr[GRCh37] 16p13.13(1,765,602_1,917,328)×1 dn	152 Kb	LIKELY PATHOGENIC	16p13.3 Deletion (Neurodevelopmental disorder with or without variable brain abnormalities, #618443)	TOP
6	AF	F	U/S: INCREASED NT	arr[GRCh37] 17p12(14,111,772_15,441,783)×1	1.33 Mb	PATHOGENIC	Hereditary neuropathy with liability to pressure palsy (#162500)	N/A
7	AF	F	PARENTAL ANXIETY	arr[GRCh37] 17q21.31(40,958,642_41,492,704)×1	534 Kb	PATHOGENIC		N/A
8	CVS	M	AMA	arr[GRCh37] 2p22.3(32,314,654_32,878,185)×1	563 Kb	PATHOGENIC		N/A
9	CVS	M	PARENTAL ANXIETY	arr[GRCh37] 3q29(195,804,728_197,760,071)×1 dn	1.95 Mb	PATHOGENIC	3q29 microdeletion syndrome (#609425)	N/A
10	AF	M	PARENTAL ANXIETY	arr[GRCh37] 3q29(195,038,425_197,837,049)×1	2.8 Mb	PATHOGENIC	3q29 microdeletion syndrome (#609425)	N/A
11	AF	M	FAMILY HISTORY	arr[GRCh37] 6q27(169,841,473_170,911,240)×1 arr[GRCh37] 7q33q36.3(136,282,120_159,128,556)×3	1.1 Mb, 22.8 Mb	PATHOGENIC		N/A
12	CVS	M	PARENTAL ANXIETY	arr[GRCh37] 15q13.3(32,065,000_32,509,926)×3	445 Kb	LIKELY PATHOGENIC	15q13.3 microdeletion syndrome (#612001)	Healthy at age of 2 years
13	CVS	M	FAMILY HISTORY	arr[GRCh37] 8p23.3p23.1(191,53_6,911,531)×1 mat arr[GRCh37] 16p13.3(96,766_5,453,898)×3 mat	6.7 Mb, 5.4 Mb	PATHOGENIC		N/A
14	CVS 2X400K	M	FAMILY HISTORY	arr[GRCh37] Xp11.23(48,542,491_48,546,333)×1	3.8 Kb	PATHOGENIC	Wiskott–Aldrich syndrome (#301000)	TOP
15	AF	M	PARENTAL ANXIETY	arr[GRCh37] Xp11.23(43,660,254_44,210,254)×1	550 Kb	PATHOGENIC		TOP
16	CVS	M	FAMILY HISTORY	arr[GRCh37] Xq28(153,387,892_153,541,289)×1	153 Kb	PATHOGENIC		N/A
17	AF	M	PARENTAL ANXIETY	arr[GRCh37] 10p15.3p14(102,539_8,286,394)×3 arr[GRCh37] 12p13.33p13.31(192,511_9,286,959)×3	8.2 Mb, 9.1 Mb	PATHOGENIC PATHOGENIC		N/A
18	CVS	F	FAMILY HISTORY	arr[GRCh37] 11q23.3q25(116,722,111_134,868,407)×3 mat arr[GRCh37] 22q11.1q11.21(17,397,498_20,402,677)×3 mat	18 Mb, 3 Mb	PATHOGENIC		N/A
19	CVS	M	U/S: CYSTIC HYGROMA	arr[GRCh37] 15q13.2q13.3(30,938,664_32,509,926)×3	1.57 Mb	LIKELY PATHOGENIC	15q13.3 recurrent region (includes CHRNA7)	TOP
20	AF	M	FAMILY HISTORY	arr[GRCh37] 15q13.3(32,037,769_32,509,926)×3	472 Kb	LIKELY PATHOGENIC	15q13.3 recurrent region (includes CHRNA7)	Healthy at 18 months
21	CVS	F	PARENTAL ANXIETY	arr[GRCh37] 15q13.3(32,037,769_32,635,959)×3	598 Kb	LIKELY PATHOGENIC	15q13.3 recurrent region (includes CHRNA7)	Healthy at 8 years
22	AF	M	FAMILY HISTORY	arr[GRCh37] 16p13.11(14,910,205_16,311,070)×3 pat	1.4 Mb	PATHOGENIC		N/A
23	AF	M	FAMILY HISTORY	arr[GRCh37] 16p13.11(14,910,205_16,311,070)×3 pat	1.4 Mb	PATHOGENIC		N/A
24	CVS	F	FAMILY HISTORY	arr[GRCh37] 16p13.11(14,910,205_16,742,212)×3	1.8 Mb	PATHOGENIC		N/A
25	CVS	M	PARENTAL ANXIETY	arr[GRCh37] 16p13.3(581,896_1,385,223)×3	803.3 Kb	LIKELY PATHOGENIC	16p13.3 duplication syndrome (#613458)	Healthy at 9 years
26	CVS	M	PARENTAL ANXIETY	arr[GRCh37] 19p13.2(10,870,008_13,372,813)×3	2.5 Mb	PATHOGENIC		TOP
27	CVS	M	FAMILY HISTORY	arr[GRCh37] 22q11.21(18,909,044_21,464,119)×3 pat	2.52 Mb	PATHOGENIC	22q11.2 microduplication syndrome (#608363)	N/A
28	CVS	M	FAMILY HISTORY	arr[GRCh37] 22q11.21(18,894,835_21,505,417)×3 pat	2.6 Mb	PATHOGENIC	22q11.2 microduplication syndrome (#608363)	N/A
29	CVS	F	FAMILY HISTORY	arr[GRCh37] 22q11.21(18,706,001_21,505,417)×3 pat	2.8 Mb	PATHOGENIC	22q11.2 microduplication syndrome (#608363)	N/A
30	CVS	F	PARENTAL ANXIETY	arr[GRCh37] 2q36.3(230,719,378_230,900,186)×3	180.81 Kb	LIKELY PATHOGENIC		Healthy
31	AF	M	BIOCHEMICAL SCREENING: POSITIVE PAPP-A	arr[GRCh37] 3p26.3p26.2(2,618,588_2,904,182)×3	286 Kb	LIKELY PATHOGENIC		TOP
32	CVS	M	PARENTAL ANXIETY	arr[GRCh37] 6p25.3(1,529,484_1,612,617)×3 dn	83 Kb	PATHOGENIC		N/A
33	AF	M	PARENTAL ANXIETY	arr[GRCh37] 14q12(29,104,072_29,238,620)×3	134 Kb	PATHOGENIC		N/A
34	AF	M	U/S: MEGA CISTERNA MAGNA & RIGHT FOOT CURLY TOE	arr[GRCh37] Xp22.33(61,529_2,625,515)×2	2.56 Mb	PATHOGENIC		N/A
35	AF	M	FAMILY HISTORY	arr[GRCh37]Xp22.33(60,701-2,881,568)×2 arr[GRCh37] Xq28(152,660,883_153,172,485)×2	2.8 Mb, 10.05 Mb	PATHOGENIC		N/A
36	CVS	F	FAMILY HISTORY	arr[GRCh37] Xq25(122,590,269_122,689,238)×3 mat	99 Kb	PATHOGENIC	Xq25 duplication syndrome (#300979)	TOP
37	CVS	M	PARENTAL ANXIETY	arr[GRCh37] Xq25(122,590,269_122,689,238)×2 mat	99 Kb	PATHOGENIC	Xq25 duplication syndrome (#300979)	TOP
38	AF	M	U/S: VENTRICULAR SEPTAL DEFECT, PERSISTENT LEFT SUPERIOR VENA CAVA	arr[GRCh37] Xq28(147,388,210_147,887,141)×2	499 Kb	LIKELY PATHOGENIC	Xq28 duplication syndrome (#300815)	N/A
39	CVS	F	FAMILY HISTORY	arr[GRCh37] Xq28(153,505,485_153,780,118)×3	275 Kb	PATHOGENIC (Phenotype expression in carrier females depends on X inactivation patterns)	Xq28 duplication syndrome (#300815)	N/A

**Table 2:** Cases with variants of uncertain significance.

A/A	Type	Sex	Indication for Testing	Molecular Karyotype (ISCN)	Size	Classification	Follow-Up
1	CVS	F	PARENTAL ANXIETY	arr[GRCh37] 16p13.13(11,619,313_11,719,980)×1	101 Kb	VUS	N/A
2	CVS	F	PARENTAL ANXIETY	arr[GRCh37] 16p13.13(11,547,069_11,719,980)×1	173 Kb	VUS	Healthy
3	CVS	M	PARENTAL ANXIETY	arr[GRCh37] 10q24.31q24.32(102,987,428_103,095,872)×3	108 Kb	VUS	Healthy
4	CVS	M	PARENTAL ANXIETY	arr[GRCh37] 15q11.2(22,833,122_23,672,681)×3	840 Kb	VUS	Healthy
5	AF	F	PARENTAL ANXIETY	arr[GRCh37] 15q11.2(22,698,522_23,689,829)×3	991 Kb	VUS	Healthy
6	AF	M	PARENTAL ANXIETY	arr[GRCh37] 16p11.2(31,196,169_31,227,836)×3	32 Kb	VUS	N/A
7	CVS	F	PARENTAL ANXIETY	arr[GRCh37] Xp22.2(12,633,166_12,984,557)×3	352 Kb	VUS	Mild developmental delay
8	CVS	M	PARENTAL ANXIETY	arr[GRCh37] Xq21.31q21.32(87,770,417_92,560,288)×2 dn	4.8 Mb	VUS	N/A
9	AF	M	U/S: TRANSPOSITION OF GREAT ARTERIES (D-TGA)	arr[GRCh37] Xq28(153,002,622_153,171,472)×2	169 Kb	VUS	Deceased after birth due to thrombophilia
10	AF	F	PARENTAL ANXIETY	arr[GRCh37] 6q26(162,799,322_163,029,668)×3 mat	230 Kb	VUS	Healthy

## Data Availability

The data are available upon request.
